# Supplementary material for: Influenza A virus resistance to 4’-fluorouridine coincides with viral attenuation in vitro and in vivo
Source: PLoS Pathog. 2024 Feb 1;20(2):e1011993. doi: 10.1371/journal.ppat.1011993 (PMC10863857; doi:10.1371/journal.ppat.1011993)
Supplement: S1 Table — Shown are coding mutations that were absent in lineages passaged in the presence of vehicle (DMSO) volume equivalents. Allele frequency in parentheses; frequency cut-off 5%; read depth cut-off 50. Bold underscore denotes mutations in RdRP subunits with >50% relative allele frequency that were rebuilt for resistance testing. (DOCX) [file ppat.1011993.s001.docx]

**S1 Table.** Whole genome sequencing of adapted virus populations after passage 9 (P9) or 10 (P10) as indicated. Shown are coding mutations that were absent in lineages passaged in the presence of vehicle (DMSO) volume equivalents. Allele frequency in parentheses; frequency cut-off 5%; read depth cut-off 50. Bold underscore denotes mutations in RdRP subunits with >50% relative allele frequency that were rebuilt for resistance testing.

| **adaptation lineage** | **M** | **HA** | **NA** | **NP** | **NS** | **PA** | **PB1** | **PB2** |
| --- | --- | --- | --- | --- | --- | --- | --- | --- |
| #1  (after P9) |  |  | V53F  (32.76%) | D72A  (5.98%)  S430N  (46.65%) | I54T  (99.43%) | M374V (46.46%) | **V285I (97.63%)** |  |
| #2  (after P9) |  |  | G11S  (5.99%)  N63D  (29.87%) | D72A  (5.71%) |  |  | **T46A**  **(99.88%)** | N71D  (10.2%)  **E180K**  **(57.39%)**  **E191K**  **(59.21%)**  M483K  (31.02%)  V667I  (22.69%) |
| #3  (after P9) |  |  |  | D72A  (5.61%) | A89E  (7.31%) | S291G  (5.28%) | D76N  (36.1%)  **M290V**  **(50.67%)**  I392V  (12.61%) | **K189R**  **(100%)** |
| #4  (after P10) |  | E85G  (98.34%) | Y66C  (12.48%) | V186I  (15.49%) | I54T  (86.94%) | E26G  (17.16%)  **S395N**  **(72.96%)** |  | **Y488C**  **(99.1%)**  **T491M**  **(69.52%)** |
| #5  (after P9) |  | A152S  (80%) |  | D72A  (6.01%)  S84N  (21.07%) | M104I  (7%) | **N222S**  **(71.63%)**  D386N  (18.68%) | **V285I**  **(99.92%)** | T16I  (9.67%)  S92A  (24.24%)  A271T  (5.37%)  D408G  (5.94%)  V414I  (32.77%)  D455Y  (32.45%) |
| #6  (after P10) |  |  | S31P  (99.94%)  P126L  (7.49%) |  |  | **M579I**  **(81.87%)** | **M339I**  **(99.97%)** | **Y488C**  **(98.5%)**  V731I  (32.77%) |
