# Supplementary material for: Influenza A virus resistance to 4’-fluorouridine coincides with viral attenuation in vitro and in vivo
Source: PLoS Pathog. 2024 Feb 1;20(2):e1011993. doi: 10.1371/journal.ppat.1011993 (PMC10863857; doi:10.1371/journal.ppat.1011993)
Supplement: S2 Table — (DOCX) [file ppat.1011993.s002.docx]

**S2 Table:** Dose response assays of recCA09 with rebuilt resistance mutations against 4’-FlU (EC_99_ with 95% CI and fold-change EC_99_ relative to parental recCA09 are shown).

| **Adaptation lineage** | **Mutation** | **EC_99_ and 95% CI** | **fold-change** |
| --- | --- | --- | --- |
| WT |  | 0.53 µM (0.18 - 1.7) | N/A |
| #1 | PB1 (V285I) | 0.96 µM (0.38 - 4.69) | 2× |
| #2 | PB1 (T46A) + PB2 (E180K, E191K) | 2.98 µM (0.73 - 19.78) | 6× |
| #3 | PB1 (M290V) + PB2 (K189R) | 9.19 µM (1.91 - 71.74) | 17× |
| #4 | PA (S395N) + PB2 (Y488C, T491M) | 7.75 µM (1.78 - 42.82) | 15× |
| #5 | PA (N222S) + PB1 (V285I) | 13.15 µM (4.65 - 44.87) | 25× |
| #6 | PA (M579I) + PB1 (M339I) + PB2 (Y488C) | 1.27 µM (0.2 - 20.28) | 2× |
| favipiravir resistant | PB1 (K229R) + PA (P653L) | 0.52 µM (x^A^ - 4.97) | 1× |

^A^lower confidence interval boundary could not be called
