# Supplementary material for: Influenza A virus resistance to 4’-fluorouridine coincides with viral attenuation in vitro and in vivo
Source: PLoS Pathog. 2024 Feb 1;20(2):e1011993. doi: 10.1371/journal.ppat.1011993 (PMC10863857; doi:10.1371/journal.ppat.1011993)
Supplement: S3 Table — (DOCX) [file ppat.1011993.s003.docx]

**S3 Table:** Dose response assays of recCA09 with rebuilt resistance mutations against favipiravir/T-705 (EC_99_ with 95% confidence CI and fold-change EC_99_ relative to parental recCA09 are shown).

| **Adaptation lineage** | **Mutation** | **EC_99_ and 95% CI** | **fold-change** |
| --- | --- | --- | --- |
| WT |  | 2.96 µM (x^A^ - 15.73) | N/A |
| #1 | PB1 (V285I) | 7.81 µM (2.21 - 34.78) | 3× |
| #2 | PB1 (T46A) + PB2 (E180K, E191K) | 5.52 µM (0.37 - 284.5) | 2× |
| #3 | PB1 (M290V) + PB2 (K189R) | 6.32 µM (0.24 - 660.6) | 2× |
| #4 | PA (S395N) + PB2 (Y488C, T491M) | 35.45 µM (x^A^ - 1521) | 12× |
| #5 | PA (N222S) + PB1 (V285I) | 11.09 µM (1.77 - 86.47) | 4× |
| #6 | PA (M579I) + PB1 (M339I) + PB2 (Y488C) | 9.23 µM (1.29 - 112.4) | 3× |

^A^lower confidence interval boundary could not be called
