# Supplementary material for: Influenza A virus resistance to 4’-fluorouridine coincides with viral attenuation in vitro and in vivo
Source: PLoS Pathog. 2024 Feb 1;20(2):e1011993. doi: 10.1371/journal.ppat.1011993 (PMC10863857; doi:10.1371/journal.ppat.1011993)
Supplement: S4 Table — (DOCX) [file ppat.1011993.s004.docx]

**S4 Table:** Dose response assays of recCA09 with rebuilt resistance mutations against NHC (parent compound of the prodrug molnupiravir; EC_99_ with 95% confidence CI and fold-change EC_99_ relative to parental recCA09 are shown).

| **Adaptation lineage** | **Mutation** | **EC_99_ and 95% CI** | **fold-change** |
| --- | --- | --- | --- |
| WT |  | 3.38 µM (x^A^ - 23.28) | N/A |
| #1 | PB1 (V285I) | 0.32 µM (x^A^ - 4.57) | 0.1× |
| #2 | PB1 (T46A) + PB2 (E180K, E191K) | 1.01 µM (0.21 - 11.52) | 0.3× |
| #3 | PB1 (M290V) + PB2 (K189R) | 2.29 µM (x^A^ - 983.6) | 0.7× |
| #4 | PA (S395N) + PB2 (Y488C, T491M) | 0.9 µM (x^A^ - 120.6) | 0.3× |
| #5 | PA (N222S) + PB1 (V285I) | 0.31 µM (x^A^ - 1.85) | 0.1× |
| #6 | PA (M579I) + PB1 (M339I) + PB2 (Y488C) | 2.61 µM (0.86 - 10.34) | 0.8× |

^A^lower confidence interval boundary could not be called
