# Supplementary material for: Influenza A virus resistance to 4’-fluorouridine coincides with viral attenuation in vitro and in vivo
Source: PLoS Pathog. 2024 Feb 1;20(2):e1011993. doi: 10.1371/journal.ppat.1011993 (PMC10863857; doi:10.1371/journal.ppat.1011993)
Supplement: S5 Table — (DOCX) [file ppat.1011993.s005.docx]

**S5 Table:** Peak virus titers and maximum growth rates of recCA09 with rebuilt resistance mutations.

| **adaptation lineage** | **mutation** | **max growth rate**  **[µ_max/h_]** | **max titer**  **[TCID_50_/ml]** |
| --- | --- | --- | --- |
| WT |  | 0.22 | 1.05×10^6^ |
| #1 | V285I | 0.19 | 6.49×10^4^ |
| #2 | T46A +E191K+E180K | 0.21 | 3.79×10^5^ |
| #3 | M290V+K189R | 0.25 | 3.1×10^5^ |
| #4 | S395N+Y488C+T491M | 0.24 | 8×10^5^ |
| #5 | N222S+V285I | 0.19 | 6.49×10^4^ |
| #6 | M579I+M339I+Y488C | 0.22 | 1.93×10^5^ |
