# Supplementary material for: Influenza A virus resistance to 4’-fluorouridine coincides with viral attenuation in vitro and in vivo
Source: PLoS Pathog. 2024 Feb 1;20(2):e1011993. doi: 10.1371/journal.ppat.1011993 (PMC10863857; doi:10.1371/journal.ppat.1011993)
Supplement: S6 Table — Polymorphisms matching resistance mutations are shown in bold. (DOCX) [file ppat.1011993.s006.docx]

**S6 Table:** Representation of candidate resistance mutations in complete and partial IAV sequences available in the NIH NCBI Virus sequence database (limit: 0.001%). Polymorphisms matching resistance mutations are shown in bold.

| **Resistance mutations adaptation: N222S; S395N; M579I** | | |
| --- | --- | --- |
| PA | complete sequences (1147) | partial sequences (96048) |
| N222S | N 99.04%  **S 0.7%**  H 0.17%  K 0.09% | N 98.549%  **S 0.094%**  H 0.036%  K 0.033%  D 0.012%  Y 0.011%  G 0.006%  E 0.001%  T 0.001% |
| S395N | S 98.61%  C 1.31%  **N 0.09%** | S 98.814%  **N 0.203%**  G 0.119%  C 0.069%  T 0.049%  H 0.009%  P 0.006%  I 0.002%  R 0.001% |
| M579I | M 99.74%  **I 0.17%**  V 0.09% | M 98.862%  **I 0.167%**  V 0.04%  T 0.006%  L 0.005% |

| **Resistance mutations adaptation: T46A, V285I, M290V, M339I** | | |
| --- | --- | --- |
| PB1 | complete sequences (1139) | Partial sequences (94791)p |
| T46A | T 99.65%  Q 0.18%  R 0.18% | T 98.926%  **A 0.001%**  G 0.002%  R 0.002%  K 0.001% |
| V285I | V 99.39%  Y 0.44%  R 0.18% | V 98.557%  A 0.002%  L 0.001% |
| M290V | M 99.39%  Q 0.44%  N 0.18% | M 98.521%  I 0.012%  T 0.005%  **V 0.003%**  E 0.001%  L 0.001% |
| M339I | **I 71.47%**  M 27.22%  V 0.7%  L 0.44%  P 0.18% | **I 71.529%**  M 25.951%  V 1.019%  L 0.011%  T 0.008%  F 0.002%  S 0.002%  K 0.001% |

| **Resistance mutations adaptation: E180K, K189R, E191K, Y488C, T491M** | | |
| --- | --- | --- |
| PB2 | complete sequences (1182) | partial sequences (96301) |
| E180K | E 99.92%  A 0.08% | E 98.346%  D 0.077%  G 0.015%  **K 0.007%**  A 0.001% |
| K189R | K 99.92%  **R 0.08%** | K 98.476%  **R 0.01%**  I 0.002%  N 0.002%  S 0.002%  M 0.001%  Q 0.001%  T 0.001% |
| E191K | E 95.44%  **K 4.48%**  G 0.08% | E 96.652%  **K 1.137%**  D 0.573%  G 0.079%  A 0.017%  R 0.012%  N 0.011%  Q 0.006%  V 0.004%  L 0.002%  M 0.001%  S 0.001%  T 0.001% |
| Y488C | Y 99.92%  F 0.08% | Y 98.665%  H 0.036%  F 0.012%  **C 0.005%**  D 0.001% |
| T491M | T 99.66%  A 0.34% | T 95.598%  A 2.039%  I 0.058%  N 0.008%  S 0.005%  **M 0.004%**  E 0.001%  P 0.001%  R 0.001%  V 0.001% |
