# Supplementary material for: Influenza A virus resistance to 4’-fluorouridine coincides with viral attenuation in vitro and in vivo
Source: PLoS Pathog. 2024 Feb 1;20(2):e1011993. doi: 10.1371/journal.ppat.1011993 (PMC10863857; doi:10.1371/journal.ppat.1011993)
Supplement: S7 Table — (DOCX) [file ppat.1011993.s007.docx]

**S7 Table:** Location and predicted effect of individual resistance mutations.

| **adaptation lineage** | **PA** | **PB1** | **PB2** | **rationale/location** |
| --- | --- | --- | --- | --- |
| #1 |  | V285I |  | Mutation adds bulk and pushes on the base of motif C which may alter the geometry of the polymerase active site. |
| #5 | N222S | V285I |  |  |
|  |  | V285I |  | Mutation adds bulk and pushes on the base of motif C which may alter the geometry of the polymerase active site. |
|  | N222S |  |  | Removes bulk from a central PA/PB1 interface that allows for changes in the interior channels of the polymerase. |
| #2 |  | T46A | E180K + E191K |  |
|  |  | T46A |  | Removes bulk and sits adjacent to motifs A and B. Might alter the position of the GDN site or alter the arrangement of RNA within the RNA cavity. |
|  |  |  | E180K + E191K | Alters structural mobility of PB2, allowing for altered loading of cap RNA into the central cavity. |
| #3 |  | M290V | K189R |  |
|  |  | M290V |  | Mutation reduces bulk and might allow for PB2 to reach further into core of RNA pol during cap addition, thus altering the interior environment of the RNA cavity. |
|  |  |  | K189R | Alters structural mobility of PB2, allowing for altered loading of cap RNA into the central cavity. |
| #4 | S395N |  | Y488C + T491M |  |
|  | S395N |  |  | Sits by the RNA binding site for template RNA loop. No direct effect on resistance, but may alter the loading of template RNA, working with the Y488C mutation to change geometry of RNA cavity. |
|  |  |  | Y488C | Located on PB2 and is positioned close to V285I when the cap binding domain of PB2 is brought towards the RNA cavity for cap priming. Cooperation with T491M to change the positioning of PB2 bound cap intermediate to allow for altered NTP selectivity through changes in RNA conformation within the interior cavity. |
|  |  |  | T491M | Gains bulk and may work in cooperation with Y488C to change the positioning of PB2 bound cap intermediate to allow for altered NTP selectivity through changes in RNA conformation within the interior cavity. |
| #6 | M579I | M339I | Y488C |  |
|  | M579I |  |  | Removes bulk from a central intersection of PA, PB1, and PB2. This may lead to altered structure of the interior cavity. |
|  |  | M339I |  | Near Motif F. Mutation may alter positioning of motif F, potentially allowing for altered selectivity. Sits on the opposite side of the polymerase compared to Y488C and might work in tangent to alter RNA channel and catalytic site geometry. |
|  |  |  | Y488C | Located on PB2 and is positioned close to V285I when the cap binding domain of PB2 is brought towards the RNA cavity for cap priming. Might work with M339I mutation. |
