# Supplementary material for: Influenza A virus resistance to 4’-fluorouridine coincides with viral attenuation in vitro and in vivo
Source: PLoS Pathog. 2024 Feb 1;20(2):e1011993. doi: 10.1371/journal.ppat.1011993 (PMC10863857; doi:10.1371/journal.ppat.1011993)
Supplement: S8 Table — (DOCX) [file ppat.1011993.s008.docx]

**S8 Table:** Recovery attempts of recCA09 with engineered combinations of independently emerged resistance mutations (genetic background: recCA09-nanoLuc).

| **virus** | **attempt 1**  **(11/15/22)** | **attempt 2**  **(11/27/22)** | **attempt 3**  **(12/10/22)** | **attempt 4**  **(12/20/22)** |
| --- | --- | --- | --- | --- |
| S395N + Y488C | no recovery | no recovery | no recovery | no recovery |
| T491M | no recovery | no recovery | no recovery | no recovery |
| S395N + T491M | no recovery | no recovery | no recovery | no recovery |
| V285I + K189R | no recovery | no recovery | no recovery | no recovery |
| N222S + M290V | no recovery | no recovery | no recovery | no recovery |
| V285I + Y488C | no recovery | no recovery | no recovery | no recovery |
| S395N + V285I + Y488C + T491M | no recovery | no recovery | no recovery | no recovery |
| WT | recovery | recovery | recovery | Recovery |
