# Supplementary figures and images for: Influenza A virus resistance to 4’-fluorouridine coincides with viral attenuation in vitro and in vivo
Source: PLoS Pathog. 2024 Feb 1;20(2):e1011993. doi: 10.1371/journal.ppat.1011993 (PMC10863857; doi:10.1371/journal.ppat.1011993)

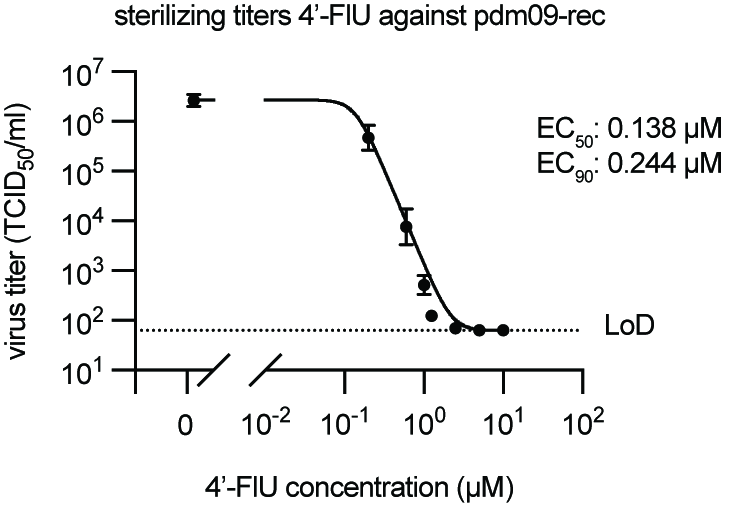

Supplement: S1 Fig — Virus yield reduction assay with recCA09. Symbols represent geometric mean ± geometric SD; line shows 4-parameter variable slope regression model. EC50 and EC90 values are given; LoD, Limit of Detection; n = 3. (TIF) [file ppat.1011993.s009.tif]

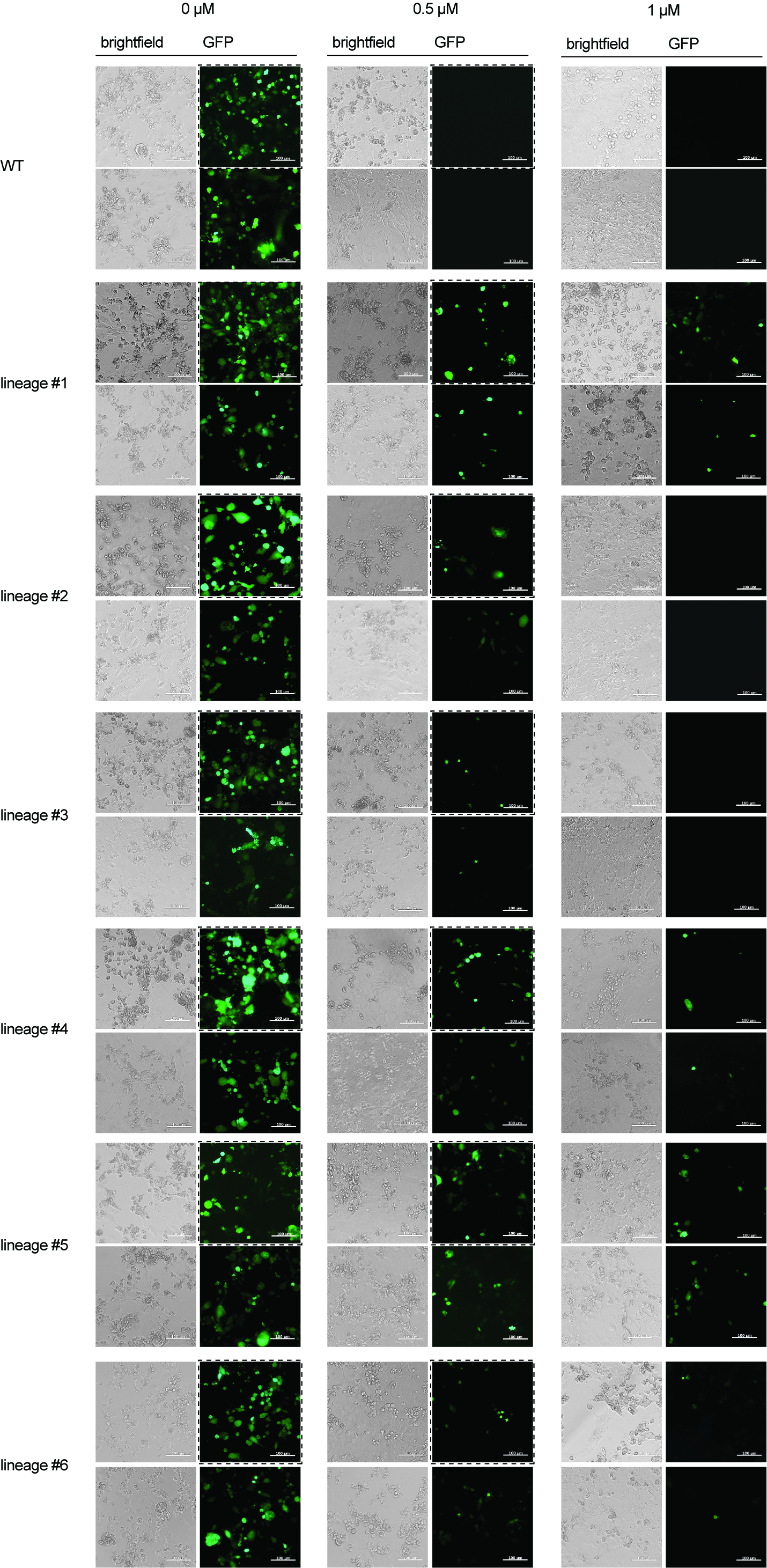

Supplement: S2 Fig — Duplicates of phase-contrast images and corresponding fluorescent images are shown for each adaptation lineage; dashed boxes denote images presented in Fig 1f; scale bar, 100 μm. (TIF) [file ppat.1011993.s010.tif]

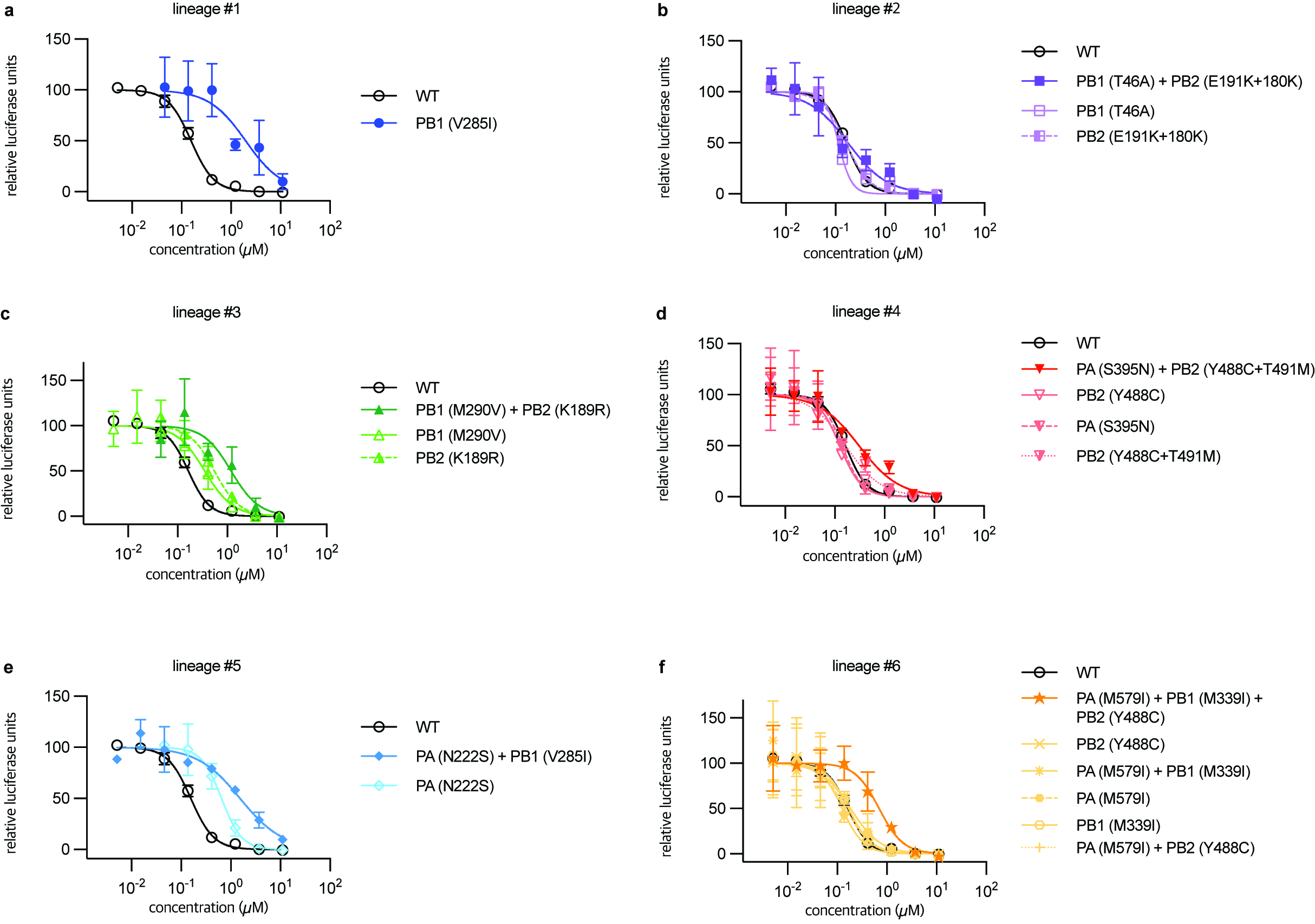

Supplement: S3 Fig — a-f) Assessment of recCA09 with mutations found in lineages 1 (a), 2 (b), 3 (c), 4 (d), 5 (e), and 6 (f). Symbols show means ± SD, lines show 4-parameter variable slope regression models. Data were normalized for samples receiving vehicle (DMSO) volume equivalents, parental recCA09 is shown in each graph; n = 3. (TIF) [file ppat.1011993.s011.tif]

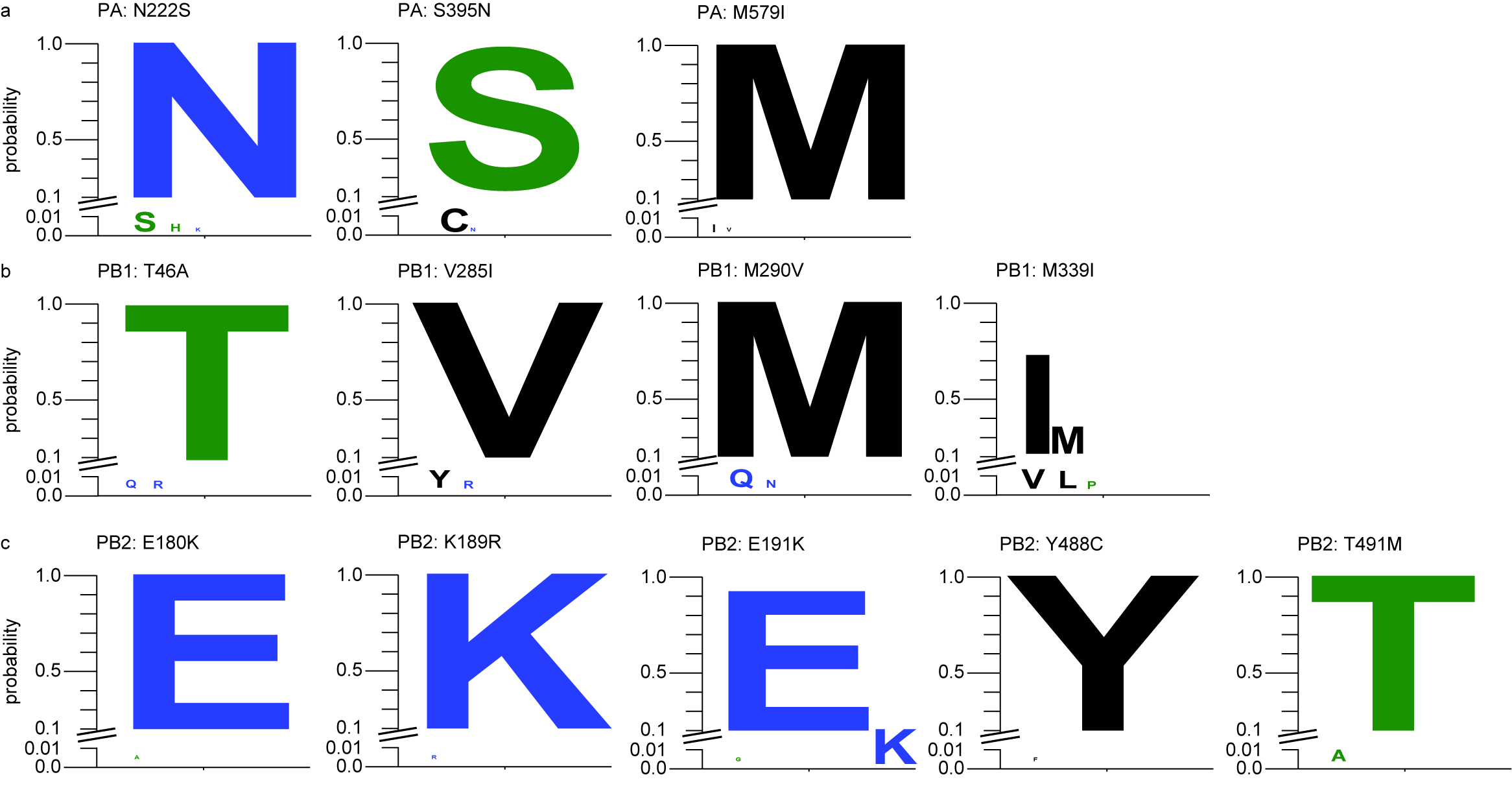

Supplement: S4 Fig — a-c) Shown are relative frequency of polymorphism at 4’-FlU resistance sites in PA (a), PB1 (b), and PB2 (c). Blue, predominant hydrophilic site chains; green, predominant neutral site chains; black, predominant hydrophobic site chains. Relative size proportional to probability of presence in the database. (TIF) [file ppat.1011993.s012.tif]

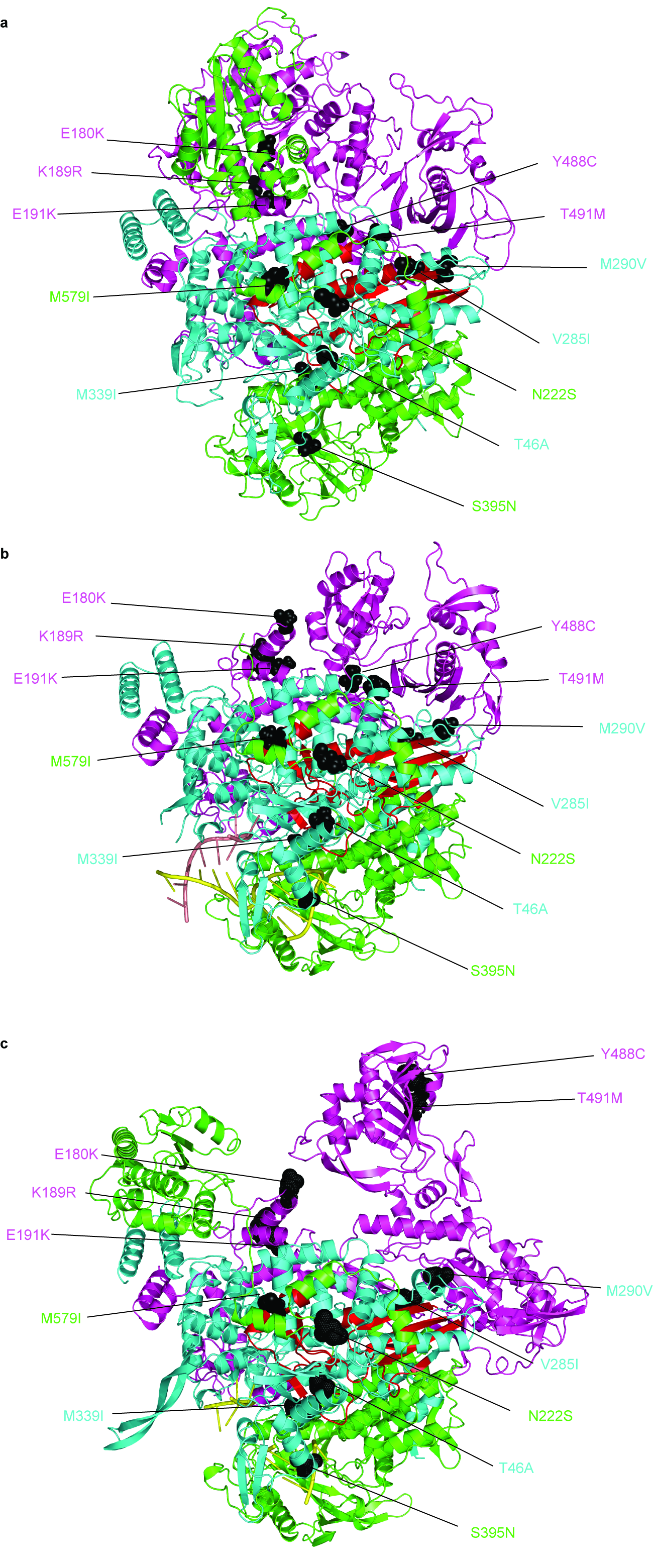

Supplement: S5 Fig — a-c) Locations of all 4’-FlU resistance mutations in a CA09 homology model based on the coordinates released for influenza C polymerase (PDBID 5d9a) (a), the 1918 H1N1 influenza A polymerase (PDBID 7ni0) (b), and a bat influenza A polymerase (4wsb) (c). Mutations are shown as black spheres, labels are color-coded by polymerase subunit; PA, green; PB1, cyan; PB2, magenta. The active site for phosphodiester bond formation of the RdRP is shown in red. Homology models were created using SWISS-MODEL, images were created using Pymol. (TIF) [file ppat.1011993.s013.tif]

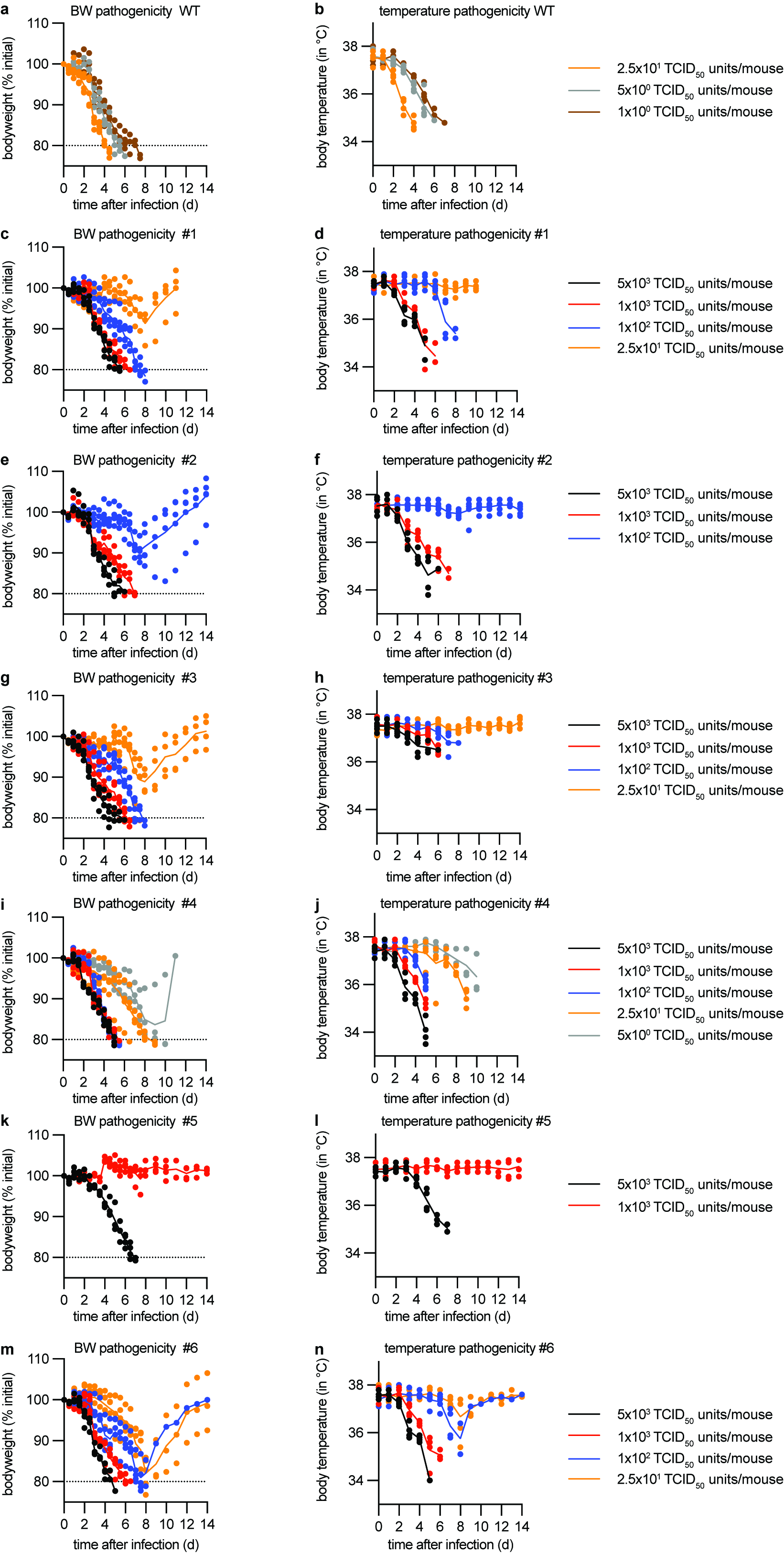

Supplement: S6 Fig — a, c, e, g, I, k, m) Bodyweight normalized to weight at the time of infection for parental recCA09 and the rebuilt resistance lineages #1–6. Dashed line, predefined humane endpoint. b, d, f, h, j, l, n) Rectal temperature measured once daily. Symbols represent individual animals, lines connect data means; n = 4–5. (TIF) [file ppat.1011993.s014.tif]

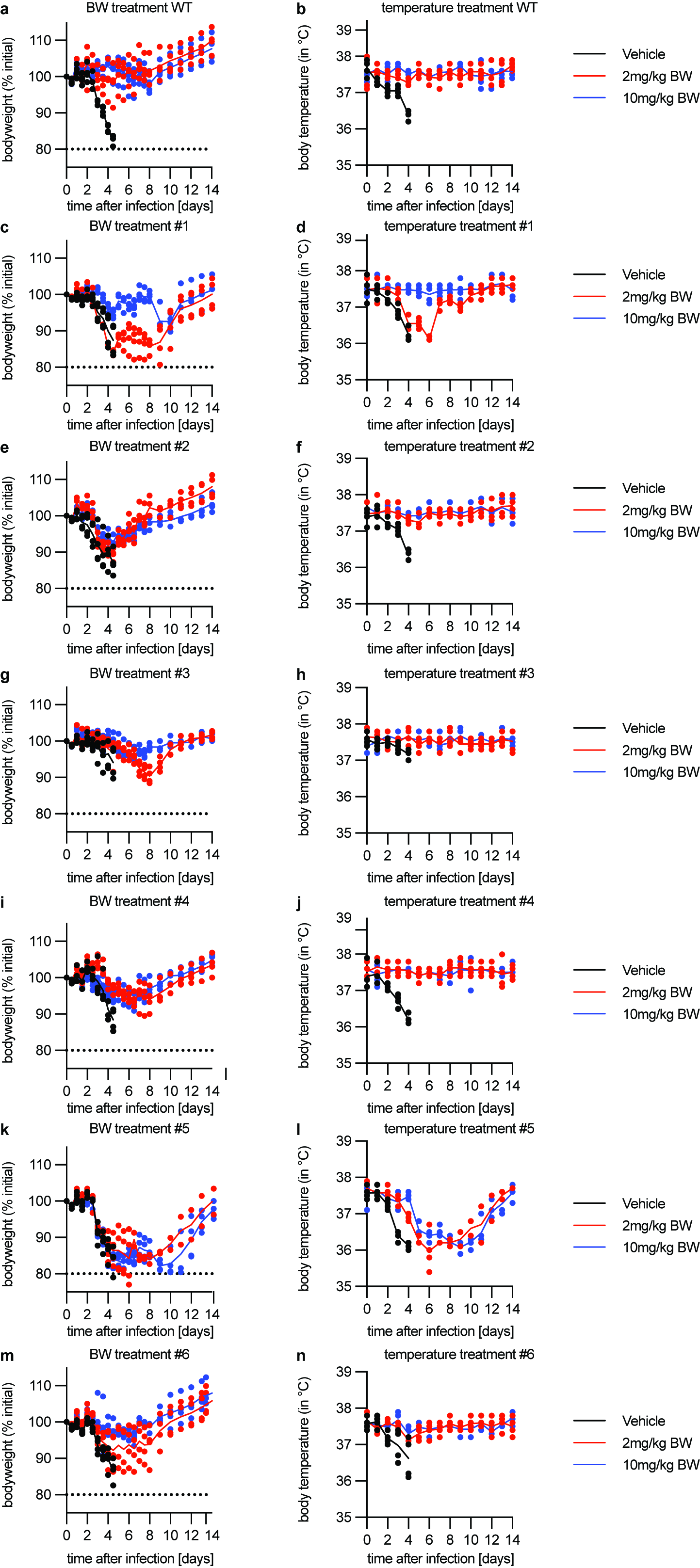

Supplement: S7 Fig — a, c, e, g, I, k, m) Bodyweight normalized to weight at the time of infection for parental recCA09 and the rebuilt resistance lineages #1–6. Dashed line, predefined humane endpoint. b, d, f, h, j, l, n) Rectal temperature measured once daily. Symbols represent individual animals, lines connect data means; n = 4. (TIF) [file ppat.1011993.s015.tif]

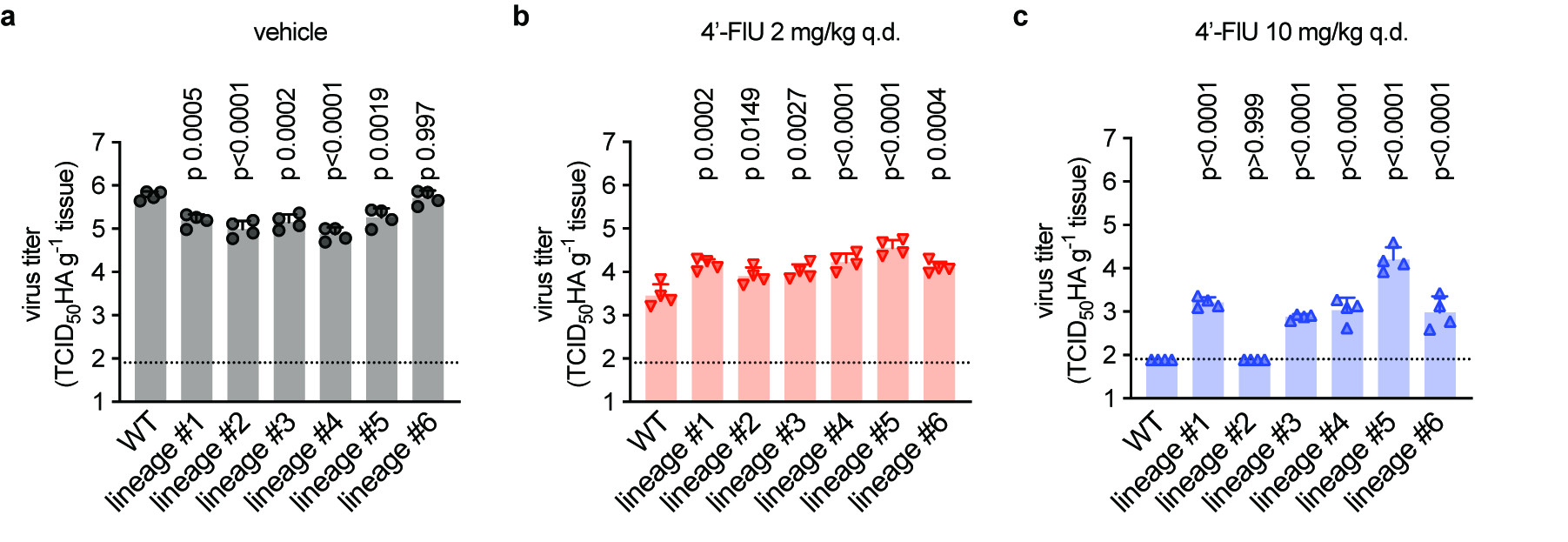

Supplement: S8 Fig — a-c) Lung virus load 4.5 dpi of animals from (Fig 4f). Results were grouped and analyzed by treatment: vehicle (a), 2 mg/kg 4’-FlU q.d. (b), or 10 mg/kg 4’-FlU (c). Symbols represent individual animals, columns show geometric means + geometric SD. Statistical analysis with 1-way ANOVA and Dunnett’s multiple comparison post-hoc test; p values are stated; n = 4. (TIF) [file ppat.1011993.s016.tif]

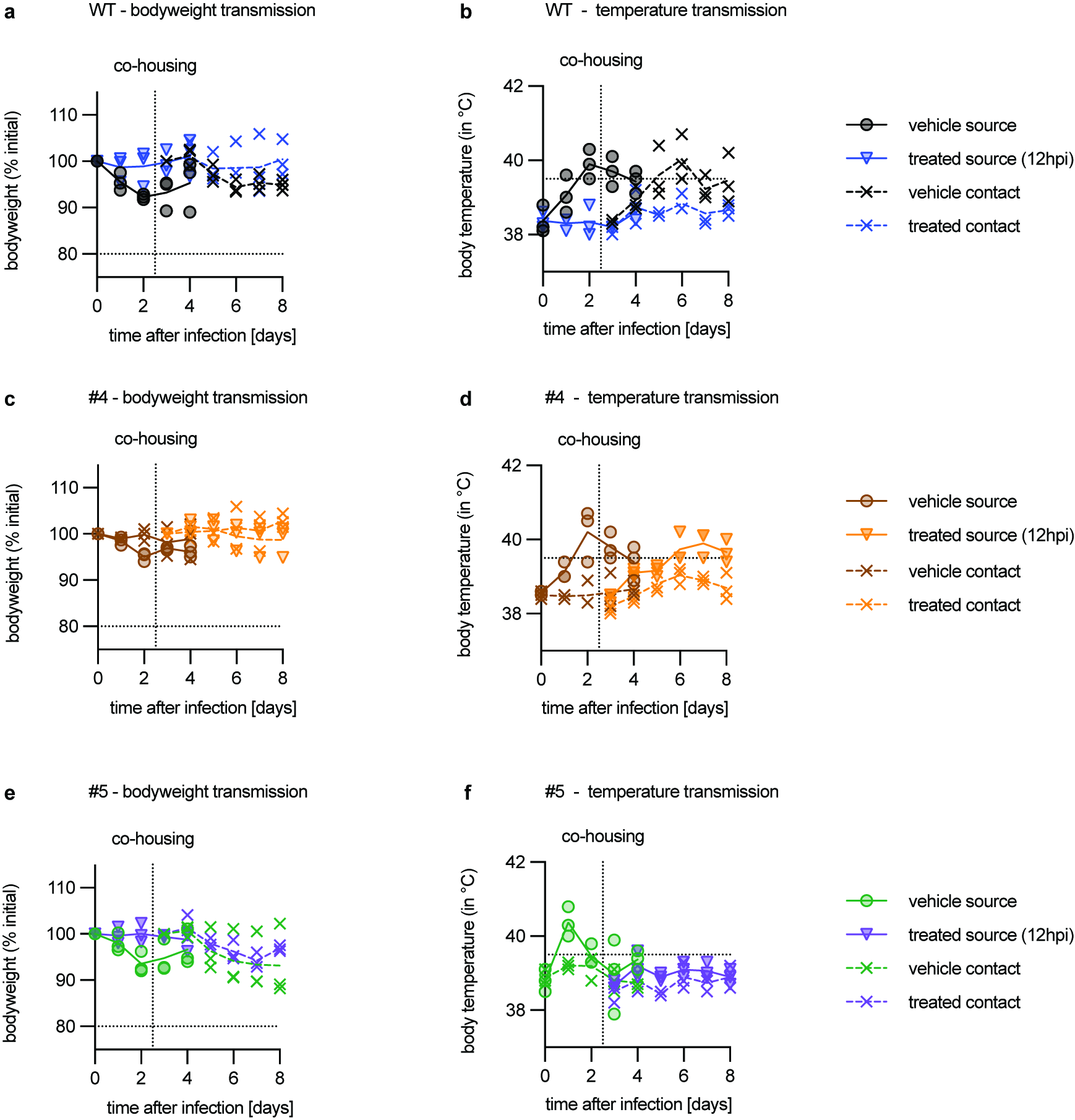

Supplement: S9 Fig — a, c, e: Bodyweight normalized to weight at the time of infection for parental recCA09 (a) and resistance lineages #4 (c) and #5 (e). Dashed line, predefined humane endpoint. b, d, f) Rectal temperature of recCA09 (b) and resistance lineages #4 (d) and #5 (f) measured once daily. Dashed line, onset of fever (39.5°C). Symbols represent individual animals, lines connect data means; n = 3. (TIF) [file ppat.1011993.s017.tif]
