# Supplementary material for: Influenza A virus resistance to 4’-fluorouridine coincides with viral attenuation in vitro and in vivo
Source: PLoS Pathog. 2024 Feb 1;20(2):e1011993. doi: 10.1371/journal.ppat.1011993 (PMC10863857; doi:10.1371/journal.ppat.1011993)
Supplement: Attachment — Submitted filename: Response to Reviews.docx [file ppat.1011993.s024.docx]

**Response to Reviews**

**Reviewer 1**

*This study provides a comprehensive exploration of the resistance mechanisms to 4’-fluorouridine (4’-FlU), a small molecule previously described as a broad-spectrum RdRp inhibitor. The prediction of antiviral resistance during pre-clinical development of a novel molecule and its potential implications on viral fitness and transmission is of a major importance to the field, since resistance to antivirals has been continuously described, especially among RNA viruses. Overall, the manuscript is well-written and I think it is very suitable for Plos Pathogens. Here are some comments and suggestions for the MS:*

Thank you very much indeed for this overall evaluation of our manuscript and the appreciation of the importance of the study for the field.

*1) Earlier studies showed that this compound has broad-spectrum activities - hence, it would be helpful to discuss and speculate its resistance profiles on other viruses.*

We had considered the impact of our influenza virus-based resistance profiling study for other viral indications of 4’-FlU under point (iii) of the Discussion (page 13 of the revised manuscript). This discussion was informed by our earlier observation (references 27 and 29) that 4’-FlU inhibits IAV RdRP by immediate chain termination, but acts as a delayed or sequence context-dependent delayed chain terminator on RSV and SARS-CoV-2 polymerases. Since our attempts to resistance profile RSV against 4’-FlU have been unsuccessful to date, we hypothesized that the influenza virus RdRP may have access to a fitness landscape of moderately increased structural flexibility in the central RdRP cavity while maintaining bioactivity, whereas tolerance of RSV polymerase to accommodate secondary structure changes may not be further expandable without loss of polymerase function. For this revision, we have expanded this discussion as requested, now speculating that, if this hypothesis is correct, an equivalent path to moderate resistance as that mapped in this study for CA09 may be available for unrelated viruses susceptible to 4’-FlU, provided the compound inhibits the polymerases of these viruses through direct chain termination.

*2) The escape mutations predominantly characterized in this study were found primarily within the RdRP subunits. Have any mutations been identified in other viral RNA segments? If such mutations have been detected, please include the corresponding data in the supplementary section.*

All adaptation lineages were submitted to whole genome sequencing after different passages. The supplementary S1Table already showed all mutations with a relative allele frequency >5% that were detected in any of the adaptation lineages, but were absent in reference virus populations that were passaged in parallel in the presence of vehicle (volume equivalent DMSO).

*3) Is there any data on the resistance-profiling of 4’-FlU using other IAV strains and influenza B? If not, the authors may need to soft the following statements such as "these results demonstrate that partial viral escape from 4’-FlU is feasible in principle, but escape mutation clusters are unlikely to reach clinical significance or persist in circulating influenza virus strains." (lines 37-39), "these results define a high genetic barrier of 4’-FlU against influenza virus escape from inhibition." (lines 94,95), and "influenza virus escape from the developmental drug 4’-FlU is mandatorily linked to viral attenuation and reduced transmission competence." (lines 361, 362).*

We apologize if we unintendedly created the impression that we meant to overstate our results. Adaptations were carried out with CA09 (pdm09 IAV) only. We have rewarded the relevant passages accordingly as recommended.

*4) Authors could define the abbreviation 4`-FIU at first appearance in the text.*

The abbreviation has been defined at first appearance, lines 5-6 of the Abstract.

*5) Line 103 - should be "grow" instead of "growth".*

Absolutely; corrected in this revision.

*6) Legend of Figure S2 should be corrected: “Repeats of microphotographs shown in (Fig. 1e)” and “dashed boxes denote images presented in Fig 1e” – It should be Fig 1f instead of Fig 1e.*

We are sorry for the mix-up. Corrected in this revision.

*7) Double check and standardize the presentations of EC_99_ and 95% CI values in the supplementary tables.*

All EC_99_ values and 95% CIs have been cross-checked and the presentation standardized (revised supplementary S2-S4 Tables).

*8) What is the rationale for choosing structural models based on influenza virus C (ICV), the 1918 H1N1 IAV, and a bat IAV? Discuss any potential limitations or assumptions associated with using these models for the study.*

We had built CA09 homology models for a range of different influenza virus polymerase structures to appreciate changes in the microenvironment of identified resistance sites, including structures of the 1918 H1N1, an H5N1, the bat IAV, and the ICV polymerase complex. The predicted spatial location of the resistance mutations were virtually identically across the ICV, H1N1, and H5N1 structures. Seeing this comment, however, we agree with the reviewer that it would have been more intuitive to feature an IAV-derived, rather than the ICV, structure in figure 3. We have revised the figure and accompanying text accordingly, now showing homology models based on the H5N1 IAV structure (PDB 6QPF).

*9) In the discussion on in vivo attenuation of resistant variants, provide more insight into the potential mechanisms behind this observation. This could include exploring whether the observed attenuation is due to reduced replication efficiency, altered host interactions, or other factors.*

We have expanded this discussion in the revised manuscript as requested, included under point (iv) of the Discussion (page 13 of the revised manuscript). Since most resistance mutations also reduced fitness compared to the genetic parent virus in cell culture (Fig 2f), we favor the view that reduced replication efficiency is the most likely reason for viral attenuation *in vivo*.

*10) Address the clinical relevance of the findings. How might the observed resistance impact the use of 4’-FlU as a potential therapeutic option?*

We have expanded the discussion of developmental impact of our work as requested, now specifying that our results “demonstrate in rodent and non-rodent animals models that CA09 influenza virus escape from the developmental drug 4’-FlU is mandatorily linked to viral attenuation and reduced transmission competence. These data suggest that 4’-FlU resistant virus populations are unlikely to reach clinical significance or persist in circulation in the field, supporting the long-term therapeutic potential of the compound.”

*Part II – Major Issues:*

*None*

*Part III – Minor Issues:*

*See Summary*

Addressed in our response to the Summary evaluation provided by this reviewer.

**Reviewer 2**

*In this manuscript by Lieber et al., they continue studies on 4’-fluorouridine (4’-FIU), which is a chain-terminating broad-spectrum nucleoside inhibitor. The focus on this study is resistance profiling using A/CA/07/2009 (H1N1) as a test system for influenza A viruses. Using a recombinant CA09 virus (CA09), they generate and then characterize six independent escape lineages. The resulting escape variants have moderate resistance to the drug. The mutations are located in proximity to the active site. Fitness of resistance variant viruses in cell culture and in mice was reported to be attenuated. Attenuation of the variants was also observed in ferrets. For example, the variants (#4 and #5) had only very low levels of virus growth in the lungs of ferrets even in the absence of the drug. Also, one of the two variants did not display contact transmission, while the other variant did. Overall, the work describes several resistance variants to the compound and demonstrates that these variants have reduced fitness in vivo. Moreover, they did detect the presence of revertants in the ferrets. This work adds to the foundation of work on the drug, encourage further development.*

Thank you very much for this overall evaluation of the importance of our study for the characterization of a promising novel antiviral candidate.

*Part II – Major Issues:*

*None*

*Part III – Minor Issues:*

*1. The recombinant CA09 virus (CA09) should be described in the Materials and Methods section under the category of Cells and Viruses.*

We apologized for the omission. In this revision, the recCA09 strains is introduced in the Cells and Viruses section of Methods with appropriate literature references.

*2. Does the recombinant CA09 virus contain mouse-adaptive mutations? Or does it contain internal genes from another virus that is more lethal in mice such as PR8? I ask this because normally, A/CA/07/2009 (H1N1) is only of moderate pathogenicity in mice. However, in Figure 4 the wild-type CA09 virus they use to infect Balb/c mice is reported to have a LD_50_ < 1 TCID_50_. This is an extremely low LD_50_ in mice for an A/CA/07/2009 virus.*

Thank you very much for pointing this out. All virus inoculum stocks used for *in vivo* studies and virus populations recovered from infected animals were subjected to whole genome sequencing. We have deposited WGS results under BioProject PRJNA1028360, and are also included in the submission package as RAVA plots. The S3 Data folder contains results for the virus populations (inoculum and viruses recovered from infected animals at study end) associated with the mouse studies.

Upon reanalysis, sequences of the recCA09 used in our study are identical to those deposited for A/CA/07/2009 (H1N1) under NCBI:txid641809, with the exception of 2 point mutations in the NP ORF (D101G and L122Q) that we had not noticed before. Both mutations were found in unrelated pdm09 H1N1 isolates also (i.e. PMID 20976194 and PMID 23958124), suggesting that they most likely existed as quasispecies in the original virus isolate that we used to generate our cDNA clones. Interestingly, PMID 20976194 associates the NP D101G substitution with mouse adaptation of closely related A/CA/04/2009 (H1N1), providing a likely explanation for the high mouse pathogenicity of the recCA09 used in our study. We have added this information to the revised Methods under the Cells and Viruses section.

*3. If the recombinant CA09 virus contains an alternative backbone or mouse-adaptive mutations, this could affect the phenotypes in ferrets. From my reading, though, it sounds like virus recovered from the resistant lineages (4 and 5) were used for the ferret experiment instead of the recombinant virus. Could the authors please clarify?*

We had stated in the legend to Fig. 5, which shows the ferret experiments, that all animals were infected with recombinant recCA09 (parent or variants derived thereof with rebuild resistance mutations). We have further emphasized the nature of the virus inoculum in the revised legend to Fig. 5 (“Schematic of the direct contact transmission study, assessing parent recCA09 (WT) and rebuilt, recombinant CA09 representing lineages #1/5 and #4/6.”).

*4. In Figure 4 (mouse experiment), panel h compares in each panel vehicle to increasing concentrations of the drug for each virus separately. And each graph performs a statistical analysis. Can they also compare the virus titers of the different viruses in three panels and perform statistical analyses? This would be one panel for vehicle comparing the viruses, one panel for 2 mg/kg drug, and one panel for 10 mg/kg drug.*

We have added the requested comparison and statistical analyses to the revised manuscript in a novel supplementary figure (S8 Fig). Subsequent supplementary figures have been renumbered accordingly.

*5. On page 3, line 62, please change “99% of seasonal H1N1 isolates was” to “99% of seasonal H1N1 isolates were”. As far as I am aware, the rule is that to determine if % is a singular or plural noun, look at the noun following it. If the next noun is a plural, use a plural verb. If the next noun is singular, use a singular verb.*

Thank you for the explanation. We have corrected the typo.

**Editorial Comments**

*[1] A letter containing a detailed list of your responses to all review comments, and a description of the changes you have made in the manuscript.*

*Please note while forming your response, if your article is accepted, you may have the opportunity to make the peer review history publicly available. The record will include editor decision letters (with reviews) and your responses to reviewer comments. If eligible, we will contact you to opt in or out.*

Included in the revised submission package.

*[2] Two versions of the revised manuscript: one with either highlights or tracked changes denoting where the text has been changed; the other a clean version (uploaded as the manuscript file).*

We have included a clean version and a compare copy with all changes highlighted in the revised submission package.

*Figure Files:*

*While revising your submission, please upload your figure files to the Preflight Analysis and Conversion Engine (PACE) digital diagnostic tool, https://pacev2.apexcovantage.com. PACE helps ensure that figures meet PLOS requirements. To use PACE, you must first register as a user. Then, login and navigate to the UPLOAD tab, where you will find detailed instructions on how to use the tool. If you encounter any issues or have any questions when using PACE, please email us at figures@plos.org.*

All figure files have been uploaded to, and have passed, the Preflight Analysis and Conversion Engine (PACE) digital diagnostic tool.

*Data Requirements:*

*Please note that, as a condition of publication, PLOS' data policy requires that you make available all data used to draw the conclusions outlined in your manuscript. Data must be deposited in an appropriate repository, included within the body of the manuscript, or uploaded as supporting information. This includes all numerical values that were used to generate graphs, histograms etc.. For an example see here: http://www.plosbiology.org/article/info%3Adoi%2F10.1371%2Fjournal.pbio.1001908#s5.*

We have included Source Data and Statistical Analysis files (S5 Data and S6 Data, respectively) in the submission package, which contain all numerical raw data and all statistical analyses associated with this study. Whole genome sequencing data have been deposited under in NCBI BioProject PRJNA1028360.

*Reproducibility:*

*To enhance the reproducibility of your results, we recommend that you deposit your laboratory protocols in protocols.io, where a protocol can be assigned its own identifier (DOI) such that it can be cited independently in the future. Additionally, PLOS ONE offers an option to publish peer-reviewed clinical study protocols. Read more information on sharing protocols at https://plos.org/protocols?utm_medium=editorial-email&utm_source=authorletters&utm_campaign=protocols*

All experimental protocols have been specified in the Methods section of this manuscript.

*References:*

*Please review your reference list to ensure that it is complete and correct. If you have cited papers that have been retracted, please include the rationale for doing so in the manuscript text, or remove these references and replace them with relevant current references. Any changes to the reference list should be mentioned in the rebuttal letter that accompanies your revised manuscript. If you need to cite a retracted article, indicate the article’s retracted status in the References list and also include a citation and full reference for the retraction notice.*

We have cross-checked the reference list. None of the references cited has been retracted. Additional references have been added to the revised manuscript in response to comment #8 of reviewer #1 and comment #2 or reviewer #2.
